# Association between parents’ country of birth and smoking risks in South Korean adolescents

**DOI:** 10.1038/s41598-022-20791-7

**Published:** 2022-10-12

**Authors:** Minah Park, Seung Hoon Kim, Fatima Nari, Bich Na Jang, Eun-Cheol Park

**Affiliations:** 1 Department of Public Health, Graduate School, Yonsei University, Seoul, Republic of Korea; 2 Institute of Health Services Research, Yonsei University, Seoul, Republic of Korea; 3 Department of Preventive Medicine, Eulji University College of Medicine, Daejun, Republic of Korea; 4 Armed Forces Chuncheon Hospital, Chuncheon, Republic of Korea; 5 Department of Preventive Medicine, Yonsei University College of Medicine, 50 Yonsei-to, Seodaemun-Gu, Seoul, 03722 Republic of Korea

**Keywords:** Health policy, Health services, Public health

## Abstract

This study aimed to determine whether significant associations exist between multicultural families and adolescent smoking risks in South Korea. Data from the Korea Youth Risk Behavior Web-based survey from 2016 to 2020 were analyzed. Participants were classified into four family types (South Korean mother–foreign father, South Korean father–foreign mother, both foreign parents, and both South Korean parents) according to their parents’ country of birth and smoking was assessed using a self-reported questionnaire. A logistic regression analysis was used to examine the significance of the associations. Overall, 194,259 participants (boys: 94,793, girls: 99,466) enrolled in this study. Adolescents whose parents were born overseas were more likely to smoke than native South Korean adolescents (boys: odds ratio [OR] = 2.61, confidence interval [CI] = 1.79–3.81, girls: OR 3.94, CI 2.42–6.43). When the mother’s country of birth was a developing country, there was an increased likelihood of girls smoking, and there was an increased likelihood of smoking among boys when the mother’s country of birth was North Korea. When both parents were born abroad, and the mother’s country of birth was a developing country, the likelihood of smoking risks among their multicultural teenage children increased. Policies and interventions need to be established and implemented to lower the smoking rate among multicultural teenagers.

## Introduction

The number of multicultural families are increasing in South Korea, as the number of marriages between South Koreans and foreigners in 2019 was 23,643 constituting 8.8% of marriages in that year^1^. Since 2010, the interracial marriage rate has been around 7–9% of the total marriages in South Korea, and from 1993 to 2018, the total number of international marriages was 592,412^2^. When the numbers of multicultural children and South Korean spouses are included, their population numbers cannot be ignored^2^. Unlike previous family forms, members of these families have different cultural backgrounds. Consequently, issues that stem from more fundamental differences, such as race and language, have arisen alongside existing family challenges^3^.

Adolescent smoking often extends into adulthood, which may lead to greater social harm^4^. According to the Tobacco in Australia, the health consequences of smoking among young people include respiratory and non-respiratory effects and dental health^5^. Teenage smokers suffer from shortness of breath and produce more phlegm compared to teenagers who do not smoke^6^. Also, teenage smokers are more likely to seek help from medical professionals due to psychological and emotional issues^7^. To improve youth’s health, abstaining from smoking is necessary^8^.

Several indicators are related to adolescent smoking, including race^9^, age^10^, depression^11^, and socioeconomic factors^12^. Among these, parental factors^13^ are known to have a great influence on smoking habits of adolescents. Being in a multicultural family in South Korea, one is likely to be exposed to these indicators. As most international marriages occur between South Korean husbands and foreign wives, most policies seek to assimilate foreign spouses into Korean culture. However, little to no attention has been paid to multicultural children^14^. As average age of multicultural children is 8.3 years^15^, necessary policies and intervention strategies must be established to ensure a stable and healthy multicultural society^16^.

A study by Jang et al.^17^ investigated the association between parents’ country of birth and depression in their offspring, while Kim et al.^18^ studied multicultural families and differential risks of suicidal behaviors. Drawing from these two studies, the purpose of this study was to investigate whether there was a significant association between parents’ country of birth and smoking status in a nationally representative sample of South Korean adolescents.

## Methods

### Data

The data used in this study were derived from the Korea Youth Risk Behavior Web-based Survey (KYRBWS), which was administered by the Korea Center for Disease Control and Prevention Agency (KDCA) each year from 2016 to 2020. As a national school-based survey, the KYRBWS is conducted annually to monitor South Korean adolescent health-related behaviors^19^. The survey was self-reported, and reliability and validity have been demonstrated^20^. The microdata (in the form of Statistical Analysis Software [SAS] files) and analytic guidelines can be downloaded from the KYRBWS website (http://www.kdca.go.kr/yhs/). The final data gathered were from 194,259 individuals.

### Ethical consideration

This study was reviewed and approved by the International Review Board of Yonsei University’s Health System (IRB number: 4-2021-1437) and adheres to the tenets of the Declaration of Helsinki. Our study did not need to address any ethical concerns because the KYRBWS is a secondary dataset that is available publicly as an anonymized data without any individual identifying information.

### Variables

The variable of interest in this study was the type of family. The KYRBWS asks each participant: “Was your father/mother born in Korea?” with response options of “Yes” or “No.” Based on the answer given, the participants were divided into four family types: South Korean mother–foreign father, South Korean father–foreign mother, both foreign parents, and both South Korean parents. In the second stage of analysis, individuals were asked, “What country were your parents born in?” with the response option as one of twelve countries, including South Korea, China, North Korea, Vietnam, Philippines, Japan, Taiwan, Mongolia, Thailand, Cambodia, Russia, Uzbekistan. It was then grouped as follows: South Korea, Japan/Taiwan, Mainland China, North Korea, and others. Japan and Taiwan were grouped together, as among the listed countries as they represent countries from advanced economies^21^.

The dependent variable was smoking status. The participants were asked the question “Have you ever smoked one or two regular cigarettes?” with response options of “Yes” or “No.” The independent variables included sex, school year, economic situation, academic grade, mother’s education level, father’s education level, alcohol use, stress, depression, suicide ideation and region. Economic situation and academic grades were each divided into three categories: good, average, and bad. The school year was divided into six categories from grades 7 through 12. Mother’s and father’s education levels were divided into three categories: middle school, high school, and university. Alcohol use, depression, and suicide ideation were divided into two categories: “Yes” or “No.” Stress was divided into three categories: “a lot,” “a little,” and “none.” Region was divided into two categories: “Metropolitan” or “Rural”. Smoking Exposure at home was divided into two categories: “Yes” and “No”.

### Statistical analysis

All analyses were conducted separately for sex in consideration of the difference in smoking patterns^22^. Chi-squared test was used to assess the difference in frequencies and proportions. Multivariable regression analysis was used to evaluate the association between a multicultural family and smoking. The results were reported using odds ratios (OR) and confidence intervals (CI). Model fitting was performed using the PROC SURVEYLOGISTIC procedure and applied weight procedures, clusters, and strata. SAS 9.4 (SAS Institute Inc; Cary, North Carolina) was used for the analysis and P-values < 0.05 were considered statically significant.

## Results

Table 1 illustrates the results of the univariate analyses that examined the association between smoking habits and the four family types (South Korean mother–foreign father, South Korean father–foreign mother, both foreign parents, and both South Korean parents) and each variable by gender.

**Table 1:** General characteristics of study participants.

	Smoking									
	Boys (n = 94,793)				P value	Girls (n = 99,466)				P value
	Yes		No			Yes		No		
	N	%	N	%		N	%	N	%	
Total (n = 194,259)	18,014	(19.0)	76,779	(81.0)		6498	(6.5)	92,968	(93.5)	
**Family type**					< 0.0001					< 0.0001
South Korean mother–Foreign father	20	(20.8)	76	(79.2)		10	(10.0)	90	(90.0)	
South Korean father–Foreign mother	145	(18.2)	650	(81.8)		80	(8.2)	894	(91.8)	
Both Foreign parents	63	(34.4)	120	(65.6)		37	(21.3)	137	(78.7)	
Both South Korean parents	17,786	(19.0)	75,933	(81.0)		6371	(6.5)	91,847	(93.5)	
**School year**					< 0.0001					< 0.0001
7th	548	(3.9)	13,681	(96.1)		205	(1.4)	14,629	(98.6)	
8th	1427	(9.5)	13,573	(90.5)		610	(3.9)	15,139	(96.1)	
9th	2553	(16.1)	13,315	(83.9)		1013	(6.0)	15,908	(94.0)	
10th	3632	(22.3)	12,638	(77.7)		1244	(7.4)	15,616	(92.6)	
11th	4594	(27.3)	12,231	(72.7)		1614	(9.3)	15,752	(90.7)	
12th	5260	(31.7)	11,341	(68.3)		1812	(10.2)	15,924	(89.8)	
**Economic situation**					< 0.0001					< 0.0001
Good	7513	(17.0)	36,582	(83.0)		2247	(5.6)	37,886	(94.4)	
Average	7760	(19.4)	32,340	(80.6)		2946	(6.2)	44,757	(93.8)	
Bad	2741	(25.9)	7857	(74.1)		1305	(11.2)	10,325	(88.8)	
**Academic grade**					< 0.0001					< 0.0001
Good	5643	(13.3)	36,690	(86.7)		1744	(4.2)	39,740	(95.8)	
Average	4787	(18.3)	21,326	(81.7)		1609	(5.3)	28,466	(94.7)	
Bad	7584	(28.8)	18,763	(71.2)		3145	(11.3)	24,762	(88.7)	
**Health condition**					< 0.0001					< 0.0001
Good	8268	(18.7)	35,894	(81.3)		2284	(5.7)	37,463	(94.3)	
Average	1938	(20.5)	7,513	(79.5)		1198	(8.1)	13,535	(91.9)	
Bad	649	(23.0)	2177	(77.0)		559	(11.6)	4258	(88.4)	
**Mother's education**					< 0.0001					< 0.0001
Middle school	414	(26.6)	1144	(73.4)		230	(13.2)	1512	(86.8)	
High school	7976	(23.6)	25,761	(76.4)		3231	(8.5)	34,643	(91.5)	
University	9624	(16.2)	49,874	(83.8)		3037	(5.1)	56,813	(94.9)	
**Father's education**					< 0.0001					< 0.0001
Middle school	568	(28.6)	1415	(71.4)		246	(12.0)	1805	(88.0)	
High school	6887	(23.6)	22,243	(76.4)		2844	(9.0)	28,762	(91.0)	
University	10,559	(16.6)	53,121	(83.4)		3408	(5.2)	62,401	(94.8)	
**Alcohol use**					< 0.0001					< 0.0001
Yes	15,050	(36.8)	25,804	(63.2)		5812	(17.1)	28,162	(82.9)	
No	2964	(5.5)	50,975	(94.5)		686	(1.0)	64,806	(99.0)	
**Stress**					< 0.0001					< 0.0001
A lot	6550	(23.0)	21,937	(77.0)		3897	(8.7)	41,008	(91.3)	
A little	10,689	(17.5)	50,436	(82.5)		2521	(4.8)	50,343	(95.2)	
None	775	(15.0)	4406	(85.0)		80	(4.7)	1617	(95.3)	
**Depression**					< 0.0001					< 0.0001
Yes	5491	(27.9)	14,203	(72.1)		3465	(11.0)	28,060	(89.0)	
No	12,523	(16.7)	62,576	(83.3)		3033	(4.5)	64,908	(95.5)	
**Suicide ideation**					< 0.0001					< 0.0001
Yes	2448	(28.6)	6126	(71.4)		1989	(13.0)	13,271	(87.0)	
No	15,566	(18.1)	70,653	(81.9)		4509	(5.4)	79,697	(94.6)	
**Region**					< 0.0001					< 0.0001
Metropolitans	16,565	(18.8)	71,778	(81.2)		5987	(6.5)	86,575	(93.5)	
Rurals	1449	(22.5)	5001	(77.5)		511	(7.4)	6393	(92.6)	
**Smoking exposure at home**					< 0.0001					< 0.0001
Yes	5935	(24.6)	18,230	(75.4)		2619	(9.6)	24,736	(90.4)	
No	12,079	(17.1)	58,549	(82.9)		3879	(5.4)	63,232	(94.6)	

Among the 194,259 participants, 94,793 were boys, and 99,466 were girls. Smoking among boys was three times higher than among girls (19.0% vs. 6.5%). When both parents were born overseas, the participants’ smoking rate was significantly higher than the average (34.4% vs. 21.3%).

Table 2 illustrates the logistic regression results stratified by sex for the association between multicultural families and smoking for all variables. When both parents were born abroad, both boys and girls adolescents had increased odds of smoking (Boys: OR 2.51 CI 1.73–3.66 Girls: OR 3.82 CI 2.34–6.23). In the case of girl participants, the odds of smoking increased in all multicultural family types.

**Table 2:** Associations between smoking and participant demographics.

Variables	Smoking			
	Boys		Girls	
	OR	95% CI	OR	95% CI
**Family type**				
South Korean mother–Foreign father	1.26	(0.66–2.40)	2.58	(1.13–5.90)
South Korean father–Foreign mother	0.99	(0.79–1.25)	1.49	(1.11–2.00)
Both Foreign parents	2.51	(1.73–3.66)	3.82	(2.34–6.23)
Both South Korean parents	1.00		1.00	
**School year**				
7th	1.00		1.00	
8th	2.25	(1.99–2.54)	2.28	(1.88–2.77)
9th	3.72	(3.30–4.18)	2.79	(2.33–3.35)
10th	4.72	(4.21–5.30)	2.99	(2.50–3.58)
11th	5.10	(4.55–5.73)	2.90	(2.42–3.47)
12th	5.65	(5.04–6.34)	2.85	(2.38–3.40)
**Economic situation**				
Good	1.00		1.00	
Average	0.85	(0.82–0.89)	0.84	(0.79–0.90)
Bad	0.91	(0.85–0.97)	0.97	(0.88–1.07)
**Academic grade**				
Good	1.00		1.00	
Average	1.21	(1.15–1.27)	1.08	(0.99–1.17)
Bad	1.87	(1.78–1.97)	1.87	(1.73–2.02)
**Mother's education**				
Middle school	1.12	(0.96–1.31)	1.35	(1.11–1.65)
High school	1.10	(1.04–1.15)	1.12	(1.04–1.20)
University	1.00		1.00	
**Father's education**				
Middle school	1.25	(1.09–1.44)	1.18	(0.97–1.43)
High school	1.10	(1.05–1.16)	1.20	(1.12–1.30)
University	1.00		1.00	
**Alcohol use**				
Yes	7.57	(7.21–7.94)	13.91	(12.66–15.29)
No	1.00		1.00	
**Stress**				
A lot	1.01	(0.91–1.12)	0.78	(0.60–1.01)
A little	0.97	(0.88–1.07)	0.73	(0.56–0.94)
None	1.00		1.00	
**Depression**				
Yes	1.38	(1.31–1.45)	1.51	(1.41–1.61)
No	1.00		1.00	
**Suicide ideation**				
Yes	1.18	(1.10–1.27)	1.53	(1.42–1.65)
No	1.00		1.00	
**Region**				
Metropolitans	1.00		1.00	
Rurals	1.06	(0.96–1.18)	0.91	(0.77–1.08)
**Smoking exposure at home**				
Yes	1.31	(1.26–1.37)	1.34	(1.26–1.43)
No	1.00		1.00	

Table 3 illustrates the logistic regression results for the subgroup analysis stratified by the depression and suicide ideation. Regardless of sex, participants who had both foreign parents showed higher odds of suicide ideation respectively (Boys: OR: 4.32 CI 1.75–10.62; Girls: OR: 4.70 CI 1.90–11.60).

**Table 3:** Subgroup analysis stratified by independent variables.

Variables	Smoking						
	Family Type						
	Both South Korean parents	South Korean mother–Foreign father		South Korean father–Foreign mother		Both Foreign parents	
	OR	OR	95% CI	OR	95% CI	OR	95% CI
**Boys**							
Depression							
Yes	1.00	2.85	(1.05–7.70)	1.22	(0.79–1.87)	2.28	(1.21–4.28)
No	1.00	0.81	(0.33–1.97)	0.92	(0.69–1.22)	2.59	(1.62–4.13)
Suicide ideation							
Yes	1.00	2.22	(0.53–9.17)	1.73	(0.94–3.20)	4.32	(1.75–10.62)
No	1.00	1.08	(0.51–2.30)	0.91	(0.71–1.18)	2.17	(1.40–3.36)
**Girls**							
Depression							
Yes	1.00	2.15	(0.53–8.71)	1.73	(0.93–3.23)	3.82	(1.55–9.40)
No	1.00	1.04	(0.49–2.23)	0.91	(0.71–1.18)	2.13	(2.13–3.29)
Suicide ideation							
Yes	1.00	2.89	(0.69–12.55)	1.37	(0.85–2.22)	4.70	(1.90–11.60)
No	1.00	2.28	(0.83–6.27)	1.53	(1.07–2.19)	3.44	(1.99–5.94)

**Figure 1:**
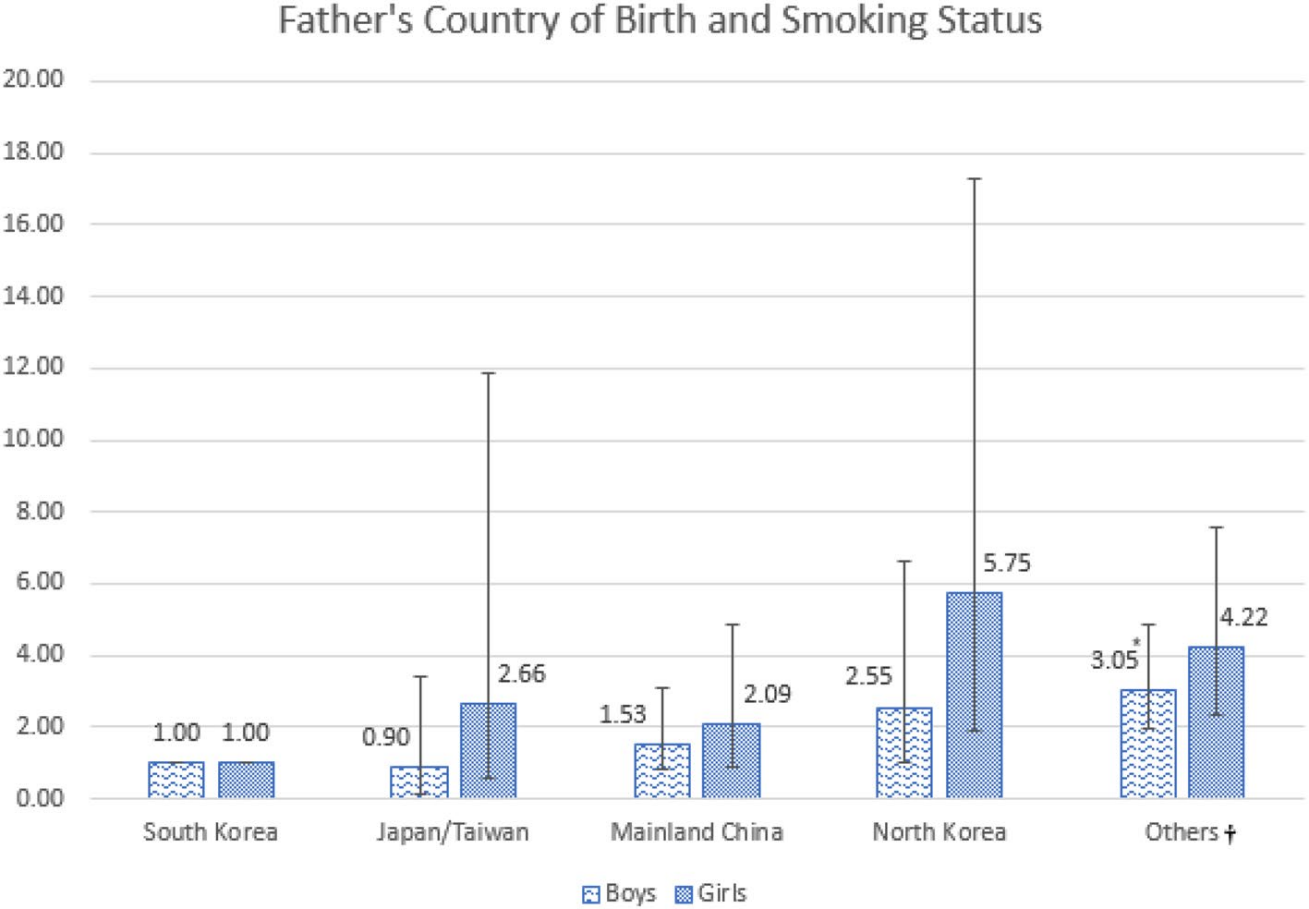
Subgroup analysis of the association between Fathers' Country of Birth and Smoking Status. *P < 0.05, **P < 0.01, *** P < 0.001. ^†^Countries including Vietnam, Philippines, Mongolia, Thailand, Cambodia, Russia and Uzbekistan.

Figure 1 illustrates the results based on the father’s country of birth and adolescents’ smoking status. Among boys, respondents whose father was born in the “Other country” group were more likely to have increased odds of smoking (Supplementary Table S3).

**Figure 2:**
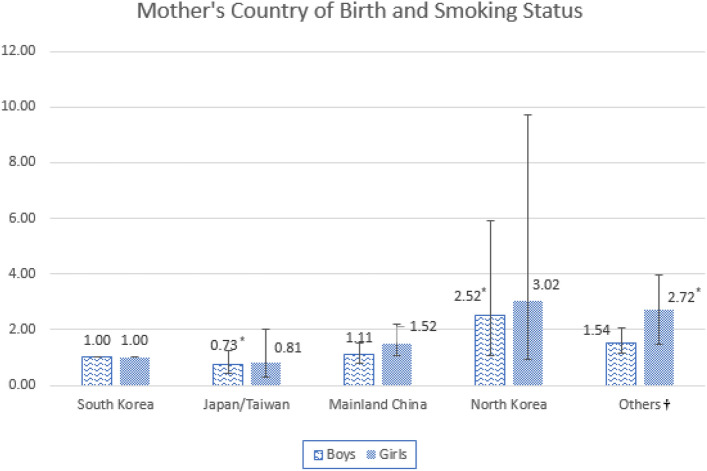
Subgroup analysis of the association between Mother's Country of Birth and Smoking Status. *P < 0.05, **P < 0.01, ***P < 0.001. ^†^Countries including Vietnam, Philippines, Mongolia, Thailand, Cambodia, Russia and Uzbekistan.

Figure 2 illustrates the results based on the mother’s country of birth and adolescents’ smoking status. In boy participants, respondents whose mother was born in North Korea were more likely to have increased odds of smoking. In girl participants, respondents whose mother was born in the “Other country” group were more likely to have increased odds of smoking (Supplementary Table S3).

## Discussion

This study aimed to identify if there is a significant correlation between multicultural families and smoking among adolescents. Our results indicate that when girl adolescents have a multicultural background, they have increased odds of smoking and boys’ adolescents only have increased odds of smoking when both parents are foreigners.

There are many aspects of the higher smoking status in multicultural adolescents compared with South Korean adolescents. One such aspect is the influence of peers on the multicultural adolescent smoking status. Peer networks and their influence have been identified as important factors, as they can engage or abstain from risk‐taking behaviors^23^. Friendships are formed on the basis of common behaviors, including smoking, and studies show that adolescents seek out groups of friends with similar attitudes and smoking behaviors^23^. In a recent study conducted among eighth-grade students in South Korea, multicultural adolescents were more likely to engage in wrongdoings than monocultural adolescents, and smoking was no exception^24^.

In addition, depending on the parent’s country of birth, this could have influenced the difference in smoking status among adolescents. Attitude toward smoking may differ, such as direct parental smoking depending on nationality^25^. Furthermore, the tendency of multicultural families with low socioeconomic status(SES) could be a reason. Especially in South Korea, married multicultural families tend to have a large age gap, are low-income, and have low educational levels^26^. In terms of income, a 2017 study by the Korean Youth Policy Institute showed that the average monthly income of multicultural households was 2.68 million won (equivalent to $2135), which is approximately 1 million won lower than most South Korean households^27^. As low SES is known to be a proxy measure for family attitudes toward the locus of control and the general value of health^28^, this could have had a large influence on smoking in multicultural adolescents.

The difference in results by sex could be due to social sanctions. In South Korea, youth smoking is viewed as a delinquent behavior, but there is a tendency to view it as a more serious act among girls^22^. In addition, smoking is considered a male characteristic, as it shows masculinity and male bonding^29^. This could be why, the smoking rate in boys was similar across the board despite the difference in the nationalities of their parents.

Children with foreign born parents were more likely to experience depression and suicide ideation. These children tend to face conflicts while growing up in different cultures their experiences can cause issues with their self-identity and values^30^. Additionally, a lack of Korean-language skills can affect multicultural adolescents. Limited Korean language ability can lead to difficulty in understanding the culture, eventually resulting in high cultural adaptation stress^31^. Similarly, a lack of Korean-language skills can decrease self-esteem, thereby increasing stress and depression^32^. Additionally, limitations in verbal communication can cause difficulties in school relationships and missed tasks presented by the school^31^. These factors can affect the mental health of adolescents, which is closely linked to smoking issues^33^.

Multicultural adolescents with foreign-born parents from a lower-income country have a higher risk of smoking than native Korean adolescents. This could be due to many reasons. It could be linked to the fact that 34% of children aged 13–15 who smoke in various forms are from Southeast Asia. Moreover, the smoking rate of adolescents aged 15 and above in both genders are 45%, which is the highest in the world. Moreover, when comparing smoking rates between South Korea (men: 34%, women: 6.7%) to other countries, smoking was higher in lower-income countries^34^. For example, countries such as Russia have the highest smoking rate in Europe among men (more than 60%) and women(more than 20%)^35^. In Uzbekistan, although the smoking rate for women was low (1.6%), it was 38.1% for men^36^. The absence of tobacco-related legal regulations in Southeast Asian countries and low-risk awareness are inferred to have been more influential among teenagers from multicultural families in Korea^37^. Further, discrimination based upon skin color, or being treated as an outcast, is a common experience for multicultural children^38^. Additionally, people from developing countries face more discrimination than those from more developed countries^39^. According to a study, people with appearances similar to Koreans are less likely to be discriminated compared to people who have different appearances^40^. Experiences of discrimination can cause negative feelings and, to erase these emotions, delinquency behaviors such as smoking are more likely^41^.

Multicultural adolescents with foreign-born parents from North Korea had a higher risk of smoking than South Korean adolescents. According to the Constitution of South Korea, Article 3 states the following: “The territory of the Republic of Korea shall consist of the Korean peninsula and its adjacent islands”^42^, which means that by law, North Korean defectors are seen as South Korean. However, while socially and culturally, North Korean defectors are compatriots with the same ethnic roots, they are also cultural minorities who have difficulties adapting to South Korean society and are sometimes discriminated against^43^, also due to political problems^44^; additionally, many North Korean defectors have to manage the consequences of traumatic experiences. According to research, the traumatic experiences from escaping North Korea include starvation, risk of being discovered, and stress from the vetting process by North Korean and Chinese border guards. The more psychological trauma they have faced, the higher the externalization and internalization problems^45^. Especially for young male North Korean defectors, the difficulty adapting appears as externalization problems, such as deviance and delinquency^46^.

This study has some limitations. First, as this was a cross-sectional survey, causalities could not be confirmed. Second, the data were self-reported by the participants. It is possible that the responses did not match the actual smoking status. Third, the KYRBWS only includes Asian countries. According to Statistics Korea^47^, the top five nationalities represented in international marriages between South Korean women and foreign men include men from the US, Australia, and Canada. Fourth, factors such as peer influence could not be observed due to limitations of data. These limitations should be considered in future studies.

Despite the limitations, this study has its strengths. First, this study used the most recent, multistage, national stratified collected data. Therefore, the results are representative of adolescents in South Korea. Second, by dividing participants by family type, this study offers new insights into the association between parents’ country of birth and adolescent smoking status.

## Conclusion

There was a correlation between the parents’ country of birth and adolescent smoking status compared to adolescents whose parents were both South Koreans. When both parents were born outside of South Korea when the mother’s country of birth was a developing country or North Korea, the likelihood of smoking increased. A targeted government policies and interventions are necessary to lower the smoking rate among multicultural adolescents in South Korea (Supplementary Tables).

## Supplementary Information

Supplementary Tables.

## Data Availability

The datasets generated and/or analyzed during the current study are available in the [Korea Youth Risk Behavior Web-based Survey (KYRBWS)] repository, (http://www.kdca.go.kr/yhs/).
