# Author Correction: Association between parents’ country of birth and smoking risks in South Korean adolescents

**DOI:** 10.1038/s41598-022-25962-0

**Published:** 2022-12-13

**Authors:** Minah Park, Seung Hoon Kim, Fatima Nari, Bich Na Jang, Eun‑Cheol Park

**Affiliations:** 1 Department of Public Health, Graduate School, Yonsei University, Seoul, Republic of Korea; 2 Institute of Health Services Research, Yonsei University, Seoul, Republic of Korea; 3 Department of Preventive Medicine, Eulji University College of Medicine, Daejun, Republic of Korea; 4 Armed Forces Chuncheon Hospital, Chuncheon, Republic of Korea; 5 Department of Preventive Medicine, Yonsei University College of Medicine, 50 Yonsei‑to, Seodaemun‑Gu, Seoul, 03722 Republic of Korea

Correction to: *Scientific Reports* https://doi.org/10.1038/s41598-022-20791-7, published online 12 October 2022

The original version of this Article contained errors in Table 1, where there was a missing value in a row under “Smoking exposure at home—Yes” in column “Girls—No—N”. The correct and incorrect values appear below.

Incorrect:

**Table:** 

	Smoking									
	Boys (n = 94,793)				*P* value	Girls (n = 99,466)				*P* value
	Yes		No			Yes		No		
	N	%	N	%		N	%	N	%	
**Total (n = 194,259)**	18,014	(19.0)	76,779	(81.0)		6498	(6.5)	92,968	(93.5)	
**Smoking exposure at home**					< 0.0001					< 0.0001
Yes	5935	(24.6)	18,230	(75.4)		2619	(9.6)		(90.4)	
No	12,079	(17.1)	58,549	(82.9)		3879	(5.4)	68,232	(94.6)	

Correct:

**Table:** 

	Smoking									
	Boys (n = 94,793)				*P* value	Girls (n = 99,466)				*P* value
	Yes		No			Yes		No		
	N	%	N	%		N	%	N	%	
**Total (n = 194,259)**	18,014	(19.0)	76,779	(81.0)		6498	(6.5)	92,968	(93.5)	
**Smoking exposure at home**					< 0.0001					< 0.0001
Yes	5935	(24.6)	18,230	(75.4)		2619	(9.6)	24,736	(90.4)	
No	12,079	(17.1)	58,549	(82.9)		3879	(5.4)	68,232	(94.6)	

The original Article has been corrected.

