# Supplementary material for: Association between parents’ country of birth and smoking risks in South Korean adolescents
Source: Sci Rep. 2022 Oct 12;12:17094. doi: 10.1038/s41598-022-20791-7 (PMC9556516; doi:10.1038/s41598-022-20791-7)
Supplement: Supplementary file 1 — Supplementary Tables. [file 41598_2022_20791_MOESM1_ESM.docx]

**Association between Parents’ Country of Birth and Smoking Risks in South Korean Adolescents**

Minah Park ^a,b^, Seung Hoon Kim^e^, Fatima Nari ^a,b^, Bich Na Jang ^d^, Eun-Cheol Park ^b,c^ *

^a^ Department of Public Health, Graduate School, Yonsei University, Seoul, Republic of Korea

^b^ Institute of Health Services Research, Yonsei University, Seoul, Republic of Korea

^c^ Department of Preventive Medicine, Yonsei University College of Medicine, Seoul, Republic of Korea

^d^ Armed Forces Chuncheon Hospital, Chuncheon, Republic of Korea

^e^ Department of Preventive Medicine, Eulji University College of Medicine, Daejun, Republic of Korea

**Supplementary Table S1. Demographic characteristics of the study population**

|  |  | **Boys** | | **Girls** | |
| --- | --- | --- | --- | --- | --- |
|  |  | **N** | **%** | **N** | **%** |
| **Family Type** | |  |  |  |  |
| South Korean mother-Foreign father | | 96 | (0.10) | 100 | (0.10) |
| South Korean father-Foreign mother | | 795 | (0.84) | 974 | (0.98) |
| Both Foreign parents | | 183 | (0.19) | 174 | (0.17) |
| Both South Korean parents | | 93,719 | (98.87) | 98,218 | (98.75) |
| **School Year** | |  |  |  |  |
| 7th |  | 14,229 | (15.01) | 14,834 | (14.91) |
| 8th |  | 15,000 | (15.82) | 15,749 | (15.83) |
| 9th |  | 15,868 | (16.74) | 16,921 | (17.01) |
| 10th |  | 16,270 | (17.16) | 16,860 | (16.95) |
| 11th |  | 16,825 | (17.75) | 17,366 | (17.46) |
| 12th |  | 16,601 | (17.51) | 17,736 | (17.83) |
| **Economic Situation** | |  |  |  |  |
| Good |  | 44,095 | (46.52) | 40,133 | (40.35) |
| Average |  | 40,100 | (42.30) | 47,703 | (47.96) |
| Bad |  | 10,598 | (11.18) | 11,630 | (11.69) |
| **Academic Grade** | |  |  |  |  |
| Good |  | 42,333 | (44.66) | 41,484 | (41.71) |
| Average |  | 26,113 | (27.55) | 30,075 | (30.24) |
| Bad |  | 26,347 | (27.79) | 27,907 | (28.06) |
| **Health Condition** | |  |  |  |  |
| Good |  | 44,162 | (46.59) | 39,747 | (39.96) |
| Average |  | 9,451 | (9.97) | 14,733 | (14.81) |
| Bad |  | 2,826 | (2.98) | 4,817 | (4.84) |
| **Mother's Education** | |  |  |  |  |
| Middle School | | 1,558 | (1.64) | 1,742 | (1.75) |
| High School | | 33,737 | (35.59) | 37,874 | (38.08) |
| University |  | 59,498 | (62.77) | 59,850 | (60.17) |
| **Father's Education** | |  |  |  |  |
| Middle School | | 1,983 | (2.09) | 2,051 | (2.06) |
| High School |  | 29,130 | (30.73) | 31,606 | (31.78) |
| University |  | 63,680 | (67.18) | 65,809 | (66.16) |
| **Alcohol Use** | |  |  |  |  |
| Yes |  | 40,854 | (43.10) | 33,974 | (34.16) |
| No |  | 53,939 | (56.90) | 65,492 | (65.84) |
| **Stress** |  |  |  |  |  |
| A Lot |  | 28,487 | (30.05) | 44,905 | (45.15) |
| A Little |  | 61,125 | (64.48) | 52,864 | (53.15) |
| None |  | 5,181 | (5.47) | 1,697 | (1.71) |
| **Depression** |  |  |  |  |  |
| Yes |  | 19,694 | (20.78) | 31,525 | (31.69) |
| No |  | 75,099 | (79.22) | 67,941 | (68.31) |
| **Suicide Ideation** | |  |  |  |  |
| Yes |  | 8,574 | (9.04) | 15,260 | (15.34) |
| No |  | 86,219 | (90.96) | 84,206 | (84.66) |
| **Region** |  |  |  |  |  |
| Metropolitans | | 88,343 | (93.20) | 92,562 | (93.06) |
| Rurals |  | 6,450 | (6.80) | 6,904 | (6.94) |
| **Smoking Exposure at Home** |  |  |  |  |  |
| Yes |  | 24,165 | (25.49) | 27,355 | (27.50) |
| No |  | 70,628 | (74.51) | 72,111 | (76.07) |

**Supplementary Table S2. Association between smoking and family types**

| **Variables** | **Smoking** | | | | | | | | | | | | | |
| --- | --- | --- | --- | --- | --- | --- | --- | --- | --- | --- | --- | --- | --- | --- |
|  | **Model 1^a,*^** | | | |  | **Model 2^b,*^** | | | |  | **Model 3 ^c,*^** | | | |
|  | **OR** | **95% CI** | | |  | **OR** | **95% CI** | | |  | **OR** | **95% CI** | | |
|  |  |  |  |  |  |  |  |  |  |  |  |  |  |  |
| **Family Type** |  |  |  |  |  |  |  |  |  |  |  |  |  |  |
| South Korean mother-Foreign father | 1.60 | (0.98 | - | 2.62) |  | 1.26 | (0.66 | - | 2.40) |  | 2.58 | (1.13 | - | 5.90) |
| South Korean father-Foreign mother | 1.11 | (0.93 | - | 1.33) |  | 0.99 | (0.79 | - | 1.25) |  | 1.49 | (1.11 | - | 2.00) |
| Both Foreign parents | 2.95 | (2.21 | - | 3.98) |  | 2.51 | (1.73 | - | 3.66) |  | 3.82 | (2.34 | - | 6.23) |
| Both South Korean parents | 1.00 |  |  |  |  | 1.00 |  |  |  |  | 1.00 |  |  |  |

^a^ Both boys and girls

^b^ Only with boys

^c^ Only with girls

^*^ Adjusted for all covariates which includes school year, economic situation, academic grade, health condition, mother’s education, father’s education, alcohol use, stress, depression, suicide ideation, region and smoking exposure at home

**Supplementary Table S3. Association between country’s of birth and smoking status.**

| **Variables** |  | **Father's country of birth** | | | |  | **Mother's country of birth** | | | |
| --- | --- | --- | --- | --- | --- | --- | --- | --- | --- | --- |
|  |  | **Smoking** | | | |  | **Smoking** | | | |
|  | **N** | **OR** | **95% CI** | | | **N** | **OR** | **95% CI** | | |
| **Boys** |  |  |  |  |  |  |  |  |  |  |
| South Korea | 94,514 | 1.00 |  |  |  | 93,815 | 1.00 |  |  |  |
| Japan/Taiwan | 35 | 0.90 | (0.25 | - | 3.18) | 187 | 0.73 | (0.42 | - | 1.27) |
| Mainland China | 112 | 1.53 | (0.85 | - | 2.76) | 377 | 1.11 | (0.81 | - | 1.54) |
| North Korea | 27 | 2.55 | (0.99 | - | 6.60) | 34 | 2.52 | (1.10 | - | 5.89) |
| Others | 105 | 3.05 | (1.93 | - | 4.83) | 380 | 1.54 | (1.14 | - | 2.07) |
| **Girls** |  |  |  |  |  |  |  |  |  |  |
| South Korea | 99,192 | 1.00 |  |  |  | 98,318 | 1.00 |  |  |  |
| Japan/Taiwan | 35 | 2.66 | (0.60 | - | 11.84) | 246 | 0.81 | (0.32 | - | 2.03) |
| Mainland China | 110 | 2.09 | (0.89 | - | 4.87) | 451 | 1.52 | (1.05 | - | 2.19) |
| North Korea | 26 | 5.75 | (1.91 | - | 17.30) | 25 | 3.02 | (0.93 | - | 9.70) |
| Others | 103 | 4.22 | (2.35 | - | 7.56) | 426 | 2.72 | (1.86 | - | 3.95) |
